# Reversal Potential of Multidrug Antibiotic Resistance in *Cutibacterium acnes* by Ethanol Extracts of Rhubarb

**DOI:** 10.1002/mbo3.70096

**Published:** 2025-10-29

**Authors:** Doudou Yang, Yu Cui, Sen Zhu, Ruoliang Wang, Haijun Xu, Guanjie Zhao, Dandan Zhang, Yinku Liang

**Affiliations:** ^1^ Collaborative Innovation Center for Comprehensive Development of Biological Resources in Qinba Mountain Area of Southern Shaanxi, Qinba Provincial Key Laboratory of Biological Resources and Ecological Environment Shaanxi Province Key Laboratory of Bio Resources, College of Biological Science and Engineering, Shaanxi University of Technology Hanzhong Shaanxi Province China; ^2^ Shaanxi Sanba Fule Limited Company, Shaanxi Province Yangling Shaanxi Province China

**Keywords:** antibiotic resistance, antimicrobial agent, *Cutibacterium acnes*, reversing antibiotic resistance, rhubarb

## Abstract

*Cutibacterium acnes* (*C. acnes*) is a major contributor to acne inflammation and exhibits significant antibiotic resistance, but research focusing on reversing this resistance is limited. Rhubarb, a natural plant with known therapeutic effects, shows potential in combating antibiotic resistance, however, no studies have been explored before. The aim of this study was to investigate rhubarb's ability to reverse antibiotic resistance in *C. acnes*. Strains 11,827 and 6919 were cultured and passaged with 0.019 μg/mL ethanol extract, and antibiotic sensitivity was monitored from passage 0 to 12. The extract effectively reversed antibiotic resistance, and was also confirmed by growth curves and oxidative markers. The impact varied across antibiotics, with the most significant reversal being erythromycin (1000‐fold), followed by clindamycin (250‐fold), and a weaker effect for tetracycline (2–4‐fold). This suggests that the extract has a stronger reversal effect on antibiotics with higher resistance. LC‐MS analysis identified flavonoids and heterocyclic compounds were may be key active components, with (‐)‐epicatechin being the most abundant and crucial for antibacterial and reversal activities. The study suggests a new strategy of using rhubarb ethanol extract as a promising acne treatment with much lower resistance, with vital advantages over conventional antibiotics. These also provide new insights into using herbal plants to combat antibiotic resistance.

## Graphical abstract

1. Ethanol extract of Rhubarb effectively reversed regular clinical antibiotic resistance, and was confirmed by growth curves and oxidative markers.

2. Reversal effects were observed with erythromycin (1000‐fold), clindamycin (250‐fold) and tetracycline (2–4‐fold).

3. The extract has stronger reversal effects on antibiotics with higher resistance.

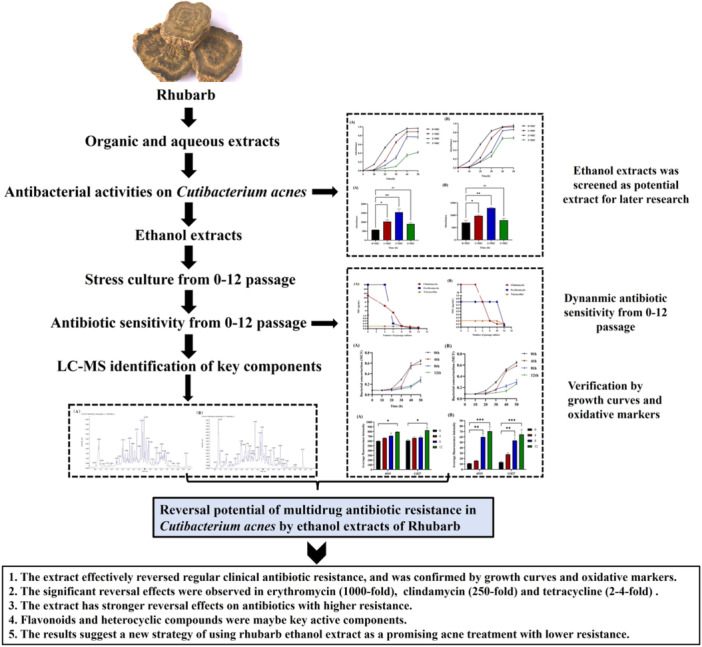

## 1 Introduction

Acne is a skin disease characterized by chronic inflammation of the hair follicle, with at least 650 million affected individuals worldwide. *Cutibacterium acnes* (*C. acnes*) is a key agent that triggers acne inflammation and immune responses (Mias et al. 2023). Therefore, inhibiting *C. acnes* with oral or topical antibacterial drugs is the most common method of acne treatment; thus, acne treatment depends mainly on antibiotics. However, clinical studies have reported a trend of increasing *C. acnes* resistance to antibiotics, including resistance to multiple common antibiotics, with the highest resistance reported to macrolides and lincomycin. Some patients with moderate to severe acne have shown poor responses to various conventional treatments, making treatment increasingly difficult (Ahle et al. 2023). To overcome the challenges of high antibiotic dependence, high dosages, and long treatment durations in the inhibition of *C. acnes*, alternative drugs with high sensitivity, low resistance, minimal side effects, and high safety are urgently needed (Yang et al. 2024).

In recent years, reversing bacterial resistance via the use of medicinal plant extracts has become a key approach in the development of antibiotic alternatives (Dong et al. 2024). It has been reported that plant extracts have advantages over single‐component antibiotics in terms of antimicrobial activity and resistance, as plant extracts contain a variety of active compounds that work synergistically to enhance antimicrobial effects (Ramalingam et al. 2024). This multitarget mechanism can effectively inhibit bacterial growth and reproduction, reducing the occurrence of resistance (Fik‐Jaskółka et al. 2024). In contrast, single antibiotics typically have a single active ingredient with a relatively simple mechanism of action, which makes it easier for bacteria to develop resistance (Fik‐Jaskółka et al. 2024). Owing to the diverse mechanisms of action of the various components of plant extracts, it is difficult for bacteria to develop resistance to all the compounds simultaneously, thereby reducing the risk of resistance development. In comparison, long‐term use of a single antibiotic often leads to bacterial resistance through mutations or gene transfers that target the mechanism of a specific antibiotic (Limboo and Singh 2024). Moreover, natural compounds in plant extracts usually have lower toxicity and fewer side effects, making them safer for long‐term use (AlSheikh et al. 2020). Therefore, plant extracts, with their multicomponent and multitarget characteristics, offer significant advantages over single antibiotics in terms of antimicrobial efficacy and resistance prevention. However, there is currently a lack of extensive research on the antimicrobial effects and resistance of medicinal plant extracts against *C. acnes*, and no studies have reported their potential to reverse antibiotic sensitivity. The roots and rhizomes of rhubarb, a plant in the *Polygonaceae* family, are used for medicinal purposes. According to traditional Chinese medicine, rhubarb has the effects of purging, clearing heat, cooling blood, detoxifying, promoting blood circulation, and resolving stasis (Wang et al. 2025; Wen et al. 2024; Zhou and Lei 2023). Pharmacological studies have shown that rhubarb has significant antitumor, laxative, antioxidant, anti‐inflammatory, and antibacterial effects. The main active ingredients in rhubarb include anthraquinones, anthraquinone ketones, tannins, and stilbenes (Wen et al. 2024). Rhubarb has shown significant therapeutic effects against acne when used topically or ingested as a single herb or in combination with other herbs(Liu et al. 2023). Previous studies have suggested that single compounds separated from rhubarb, including emodin and rhein, effectively treat acne (Liu et al. 2023). However, no studies on the effects of the crude rhubarb extract and its ability to reverse antibiotic resistance have yet been reported.

Considering the previous clinical applications of rhubarb for acne and the pressing need for alternative acne therapies, the aim of this study was to investigate the inhibitory effects of the crude extract of rhubarb on *C. acnes*, with a particular focus on its potential to reverse antibiotic resistance. The antibacterial activities of various solvent extracts were assessed through antibacterial assays to identify the most effective extract. To determine the active components of the extract, chemical analysis via HPLC‒MS/MS was conducted. *C. acnes* was then cultured with low concentrations of the compounds with the lowest minimum inhibitory concentration (MIC) values, and the changes in *C. acnes* antibiotic sensitivity after multiple passages were dynamically monitored. This study may offer new perspectives on acne prevention and treatment via a potential resistance‐lowering clinical strategy that can fully or partially replace antibiotics to address current challenges with acne treatment. Additionally, this study provides a novel foundation for studying the role of herbs in reversing antibiotic resistance, particularly highlighting the advantages of crude plant extracts, which may exert synergistic effects from multiple pharmacological components, unlike single antibiotics with a single active ingredient.

## 2 Materials and Methods

### 2.1 Materials

The dried roots of *Rhubarb* were provided by Shaanxi Sanba Fule Limited Company and were identified as *Rheum officinale Baillon* by Doudou Yang from Shaanxi University of Technology. The authentication ID was RH20230110YDD. The *C. acnes* strains ATCC6919 and ATCC11827 were provided by Warner Bio Co. Ltd., stored at −20°C and recovered before the experiment. Ethanol, petroleum ether, methanol, dimethyl sulfoxide, Tween 80, PI staining solution, anhydrous *l‐cysteine* hydrochloride, sodium acetate trihydrate, sodium chloride and other reagents were purchased from BBI Life Sciences Corporation Company.

### 2.2 Bacteria Cultivation

The medium formula of the culture medium was as follows: beef extract 10 g, yeast extract 3 g, tryptone 10 g, sodium chloride 5 g, anhydrous sodium acetate 3 g, anhydrous glucose 5 g, soluble starch 1 g, L‐cysteine hydrochloride 0.5 g, deionized water 1000 mL, 20 g agar was added to the solid medium, but agar was not added to the liquid medium. The pH was adjusted to 6.8 with 2% sodium hydroxide, then it was sterilized at 121°C for 20 min, and was cooled down to room temperature. The *C. acnes* was inoculated in the culture medium, and then cultured in the anaerobic bag at 37°C for 48 h in the incubator (Liu et al. 2023).

### 2.3 Preparation of the Rhubarb Extract

The dried *rhubarb* roots were chopped into small pieces and placed in a grinder. Rhubarb sample of 10 g was mixed with 100 mL of solvents in a round‐bottom flask. The round‐bottom flask was placed in a water bath at 80°C for extraction under reflux for 30 min, and this process was repeated 2 more times (Li et al. 2024). The filtrates extracted from the first and second extraction were combined together and put into a new round‐bottom flask and concentrated to 10% of the original volume at 60°C on a rotary evaporator, and the solvents were removed when concentrating. The concentrate was finally dried to constant weight using a vacuum freeze dryer (Janik et al. 2023). These extracts were stored separately at −20°C (Khatun et al. 2023). The extracts of ethanol, methanol, petroleum ether and water were prepared according to the above method separately.

### 2.4 Growth Inhibition Experiment

A bacterial suspension (200 μL, 10^6^ CFU/mL) was plated on solid medium. Then, sterilized antibiotic susceptibility paper discs with diameters of 6 mm were placed uniformly on the solid plates. A disc in the center with sterilized pure water were served as control. Finally, 10 μL of the diluted extract in water with concentration of 100 mg/mL extract was added to the other discs for incubation at 37°C for 48 h (Yang et al. 2019).

### 2.5 Determination of the Minimum Inhibitory Concentration (MIC) and Minimum Bactericidal Concentration (MBC)

The MIC was determined via an improved method based on the CLSI standard and Suman Tiwari's research (Belanger and Hancock 2021; Kadeřábková et al. 2024; Tiwari et al. 2023). The minimum concentration with at least a 20% reduction in the OD_600nm_ absorbance value was determined using a microplate reader compared with that of the negative control group without drugs by microdilution method in a 96‐well plate (Janik et al. 2023). MBC was determined on the basis of MIC determination, 20 μL of cultured liquid in MIC determination were taken and coated on the solid culture medium, and the colony growth was observed after further culture. The minimum drug concentration for sterile colony growth on solid medium was recorded as MBC. The *rhubarb* ethanol extract (50 mg/mL) was added into a 96‐well plate via the double dilution method to adjust the final concentration of the extract to 0.012−25 mg/mL. First, 100 μL of rhubarb extract was added to 100 μL of the liquid culture medium described in the method part, and 100 μL of this mixture was added to the next well and serially diluted with the culture medium until the last well was reached. Additionally, 50 μL of 10^6^ CFU/mL bacteria was added to each well, with three replicates in each group, and the OD_600nm_ value was measured using a microplate reader. The 0 MIC group, which was cultured without drugs, was used as a negative control.

### 2.6 Stress Culture and Passage on *C. acnes* of Rhubarb Extract

The rhubarb ethanol extract was added to the culture medium at a final concentration of 0.019 μg/mL. Subculture was performed every 48 h for 12 passages at 37°C, and the MICs of antibiotics commonly used for the clinical treatment of acne (clindamycin, erythromycin, tetracycline hydrochloride, and tetracycline) were measured at generations 0, 4, 8, 10, and 12 according to Haibo Wang et al. (Sun et al. 2023; Wang et al. 2021).

## 3 Bacterial Growth Curve

### 3.1 Bacterial Growth Curve in the Presence of the Rhubarb Extract

The extracts at concentrations of 1×MIC, 2×MIC, and 3×MIC were added to test tubes containing liquid culture medium and the prepared bacterial suspension and incubated at 37°C. Then, a 1 mL sample was removed every 10 h to measure the OD_600nm_ value. The growth curve was plotted on the basis of the OD_600nm_ value (Liang et al. 2020).

### 3.2 Bacterial Growth Curve in the Presence of Antibiotics After Passage

The strains at passages 0, 4, 8, and 12 were cultured with tetracycline‐containing culture medium at ½× MBC. A total of 10 mL of culture medium was added to a 50 mL centrifuge tube, 100 μL of each strain at a concentration of 10^6^ CFU/mL was added, and 3.9 mL of tetracycline at a concentration of 50 mg/mL was added to achieve a final tetracycline concentration of 3.25 μg/mL. The data were measured every 10 h over a total of 50 h of cultivation, and a time‒concentration growth curve was constructed (Sun et al. 2023).

### 3.3 Effect of Rhubarb Extract on Cell Permeability

The C. *acnes* suspension (1 × 10^6^ CFU/mL) was centrifuged at 6000 rpm for 10 min. The bacterial cells were collected and washed 3 times with PBS (pH 5.8). The cells were resuspended in PBS containing 0.01% Tween 80 to achieve a final concentration of 10^6^ CFU/mL. The rhubarb extract was added to the EP tube containing the suspended bacteria so that the final concentration of the rhubarb extract in the solution was 0×MIC, 1×MIC, 2×MIC, or 3×MIC, and each group had three replicates. The 0×MIC mixture was used as the control group and cultured at 37°C, and the samples were collected every 10 h and centrifuged at 8000 rpm for 5 min. The OD_260nm_ value of the supernatant was measured (Liang et al. 2020).

### 3.4 Determination of Cell Membrane Integrity

C. *acnes* (1 × 10^6^ CFU/mL) was incubated in culture medium supplemented with rhubarb extract (0×MIC, 1×MIC, 2×MIC, or 3×MIC) at 37°C for 24 h. After incubation, a viability stain solution containing a combination of propidium iodide (PI) and fluorescein diacetate (FDA) was prepared. After the cells were washed with PBS, they were stained with 5 μg/mL PI and 5 μg/mL FDA. FDA fluorescent probe (1 mL, 1 mg/mL) was added to 1 mL of bacterial suspension. After incubation at 37°C for 15 min, 1 mL of a 1 mg/mL PI fluorescent probe was added, and the mixture was left to react in the dark at room temperature for 15 min. The fluorescence value was measured with a Hitachi F‐4500 fluorescence spectrophotometer. The excitation wavelength was 533 nm, and the emission wavelength was 615 nm (Liang et al. 2020).

### 3.5 Detection of ROS in the Bacteria

DCFH‐DA1 was used as a probe, and the content of reactive oxygen species (ROS) was determined using an Aladdin reactive oxygen kit. The probe was added to the bacterial culture medium according to a specific dilution ratio (1:1000), and the final concentration of the probe was 10 μM. One milliliter of probe was added to 1 mL of bacterial suspension (1 × 10^6^ CFU/mL) and incubated at 37°C for 20 min. After incubation, the samples were washed with PBS to remove unabsorbed probes. The mixture was subsequently excited with a 488 nm laser and detected at 535 nm with a fluorescence spectrophotometer (Biswal et al. 2025).

### 3.6 Detection of DNA Damage in the Strains

Bacterial cultures at 0, 4, 8, and 12 generations were adjusted to 1 × 10^6^ CFU/mL, and the PI was adjusted to 50 μg/mL. The bacterial mixture was centrifuged and washed twice with PBS (pH 7.4). Tetracycline at a concentration of 10 μg/mL was added to a tube with 1 mL of bacterial mixture, and 1 mL of tetracycline, 1 mL of PI, and 1 mL of sterile water were mixed and then incubated for 30 min in the dark. The mixture was subsequently excited with a 525 nm laser and detected at 615 nm with a fluorescence spectrophotometer (Biswal et al. 2025).

### 3.7 Identification of the Chemical Components of the Ethanol Extract via Liquid Chromatography‒Mass Spectrometry

Sample preparation and extraction: First, 150 μL of extraction solution (ACN:methanol = 1:4, V/V) containing an internal standard was added to 150 μL of sample. The sample was then vortexed for 3 min and centrifuged at 12,000 rpm for 10 min at 4°C. A 250 μL aliquot of the supernatant was collected, incubated at −20°C for 30 min, and then centrifuged at 12,000 rpm for 3 min at 4°C. Finally, 200 μL aliquots of the supernatant were subjected to LC‒MS analysis. HPLC conditions: One aliquot was analyzed in positive ion mode and eluted from a T3 column (Waters ACQUITY Premier HSS T3 Column, 1.8 µm, 2.1 × 100 mm) using 0.1% formic acid in water as solvent A and 0.1% formic acid in acetonitrile as solvent B with the following gradient: 5% to 20% B over 2 min, increase to 60% B over 3 min, increase to 99% B over 1 min, hold at 99% B for 1.5 min, return to 5% B over 0.1 min, and hold at 5% B for 2.4 min. The analytical conditions were as follows: column temperature, 40°C; flow rate, 0.4 mL/min; and injection volume, 4 μL. Another aliquot was analyzed in negative ion mode after separation with the same elution gradient described above. MS data acquisition was performed in information‐dependent acquisition (IDA) mode using Analyst TF 1.7.1 software (Sciex, Concord, ON, Canada). The source parameters were as follows: ion source gas 1 (GAS1), 50 psi; ion source gas 2 (GAS2), 50 psi; curtain gas (CUR), 25 psi; temperature (TEM), 550°C; declustering potential (DP), 60 V in positive ion mode and −60 V in negative ion mode; and ion spray voltage floating (ISVF), 5000 V in positive ion mode and −4000 V in negative ion mode. The TOF–MS scan parameters were set as follows: mass range, 50–1000 Da; acquisition time, 200 ms; and dynamic background subtraction, on. The product ion scan parameters were set as follows: mass range, 25–1000 Da; acquisition time, 40 ms; collision energy, 30 V in positive ion mode and −30 V in negative ion mode; collision energy spread, 15; resolution, UNIT; charge state, 1‐to‐1; intensity, 100 cps; exclusion of isotopes within 4 Da; mass tolerance, 50 ppm; and maximum number of candidate ions to monitor per cycle, 18. A total of 50 compounds with high scores and relatively high content ratios (greater than 0.1%) are shown in the table.

### 3.8 Statistical Analysis

All the experiments were conducted in triplicate, and the data are presented as the means ± standard deviations (SDs). Statistical analysis was performed via one‐way ANOVA followed by Tukey's multiple comparison test. A *p*‐value of < 0.05 was considered to indicate statistical significance.

## 4 Results and Discussion

### 4.1 Screening Rhubarb Extracts Prepared Using Different Solvents for Their Inhibitory Activity Against *C. acnes*

The inhibition zone and MIC of the methanol extract, ethanol extract, petroleum ether extract, and water extract, each diluted to 100 mg/mL, were determined. As shown in Table 1, all the extracts (methanol, ethanol, petroleum ether, and water) exhibited varying degrees of antibacterial activity against *C. acnes* strains 11827 and 6919. Among these extracts, the water and ethanol extracts had the most pronounced inhibitory effects. These results suggest that water and ethanol may be more effective solvents for extracting bioactive compounds that target *C. acnes* from rhubarb. Notably, the ethanol extract had the lowest MIC values, 0.15 μg/mL for strain 11827 and 0.019 μg/mL for strain 6919, indicating that the *C. acnes* strains were particularly sensitive to this extract. On the basis of these data, the ethanol extract was chosen for further experiments to assess its potential antibacterial mechanism.

**Table 1:** Diameter of inhibition zone and MIC value of extract of rhubarb (100 mg/mL).

Extract of rhubarb	Diameter of inhibition zone (mm) MIC value (μg/mL)			
	11,827	6919	11,827	6919
Ethanol extract	11.61 ± 1.13	9.92 ± 0.22	0.15	0.02
Methanol extract	8.47 ± 0.49	8.59 ± 0.52	0.30	0.15
Petroleum ether extract	10.74 ± 0.67	8.53 ± 0.77	1.22	1.22
Water extract	14.81 ± 0.30	9.40 ± 0.94	0.30	0.60

*Note:* Data of diameter of inhibition zone are presented as mean ± SD. The bacterial strains of *Cutibacterium acnes* are ATCC 11827 and 6919.

### 4.2 Dynamic Growth Curve of *C. acnes* in the Presence of the Rhubarb Ethanol Extract

**Figure 1:**
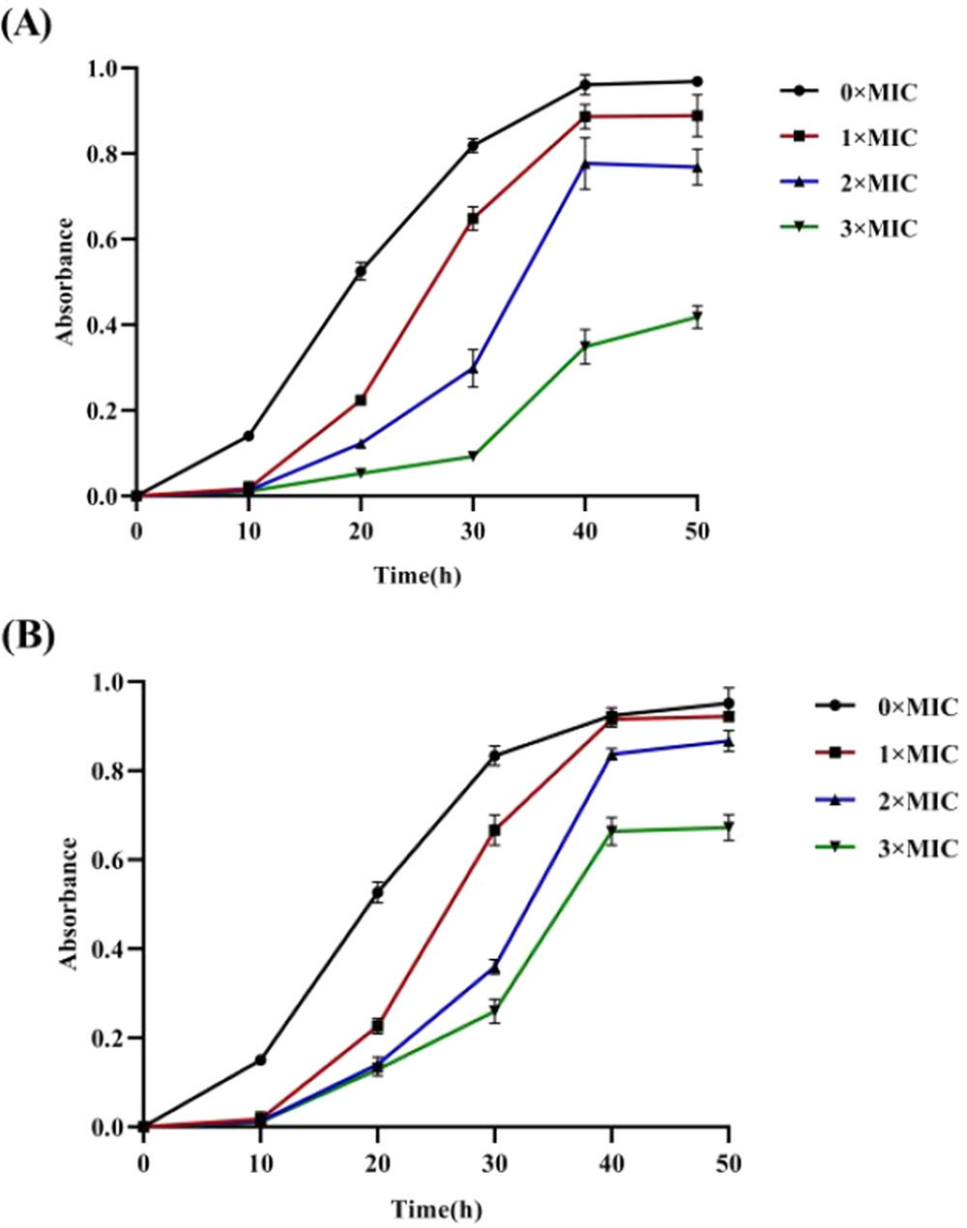
The dynamic growth curve of *C. acnes* under rhubarb ethanol extract at 1MIC, 2MIC, and 3MIC concentrations analyzed at 600 nm. (A) Growth curve of strain 6919. (B) Growth curve of strain 11,827.

To verify the antibacterial effect of the rhubarb extract and elucidate its mechanism of action, the growth of *C. acnes* in the presence of various concentrations of the ethanol extract was monitored. Ethanol extract concentrations of 0.15 and 0.019 mg/mL were chosen on the basis of the MIC values (1×MIC), and higher concentrations corresponding to 2×MIC and 3×MIC were also tested. In the untreated control group, *C. acnes* displayed typical S‐type bacterial growth, which was consistent with previous studies. In contrast, the experimental groups showed significant inhibition of bacterial growth, with more significant effects at higher concentrations. As shown in Figure 1A,B, at 1×MIC, bacterial growth was noticeably reduced compared with that of the control group, indicating the initial antibacterial activity of the extract. At 2×MIC, the inhibitory effect was even stronger, and the growth of *C. acnes* was further suppressed. Interestingly, at 3×MIC, the growth curve tended to flatten, with the absorbance value at this concentration being significantly lower than that of the other treatment groups, suggesting a potential plateau effect, where higher concentrations of the ethanol extract did not further increase bacterial inhibition. Moreover, when the bacteria were cultured under suitable conditions, on the basis of the absorbance values, the bacteria in the control group started to grow, but those in the experimental groups started to grow slightly only after 10 h and grew much more slowly than the bacteria in the control group did. When the samples were cultured for 20 h, there was less growth in the experimental groups than in the control group; however, there were notable differences among the three concentrations in the experimental groups, indicating that the inhibitory effect was concentration dependent. Bacterial growth accelerated from 10 to 40 h. After 40 h, the bacterial growth stabilized at the peak level. This finding shows that the effect of the inhibitor differed with increasing concentration. Higher concentrations of the ethanol extract strongly inhibited bacterial growth, but the low concentration of the ethanol extract also resulted in certain antibacterial activity. Therefore, the above results indicated that the rhubarb ethanol extract not only had an inhibitory effect on *C. acnes*, which exhibited dynamic growth typical of other antibacterial drugs, but also had an obvious concentration‐dependent effect.

## 5 Physical Damage to *C. acnes* Caused by the Rhubarb Ethanol Extract

### 5.1 Effect on Cell Membrane Permeability

**Figure 2:**
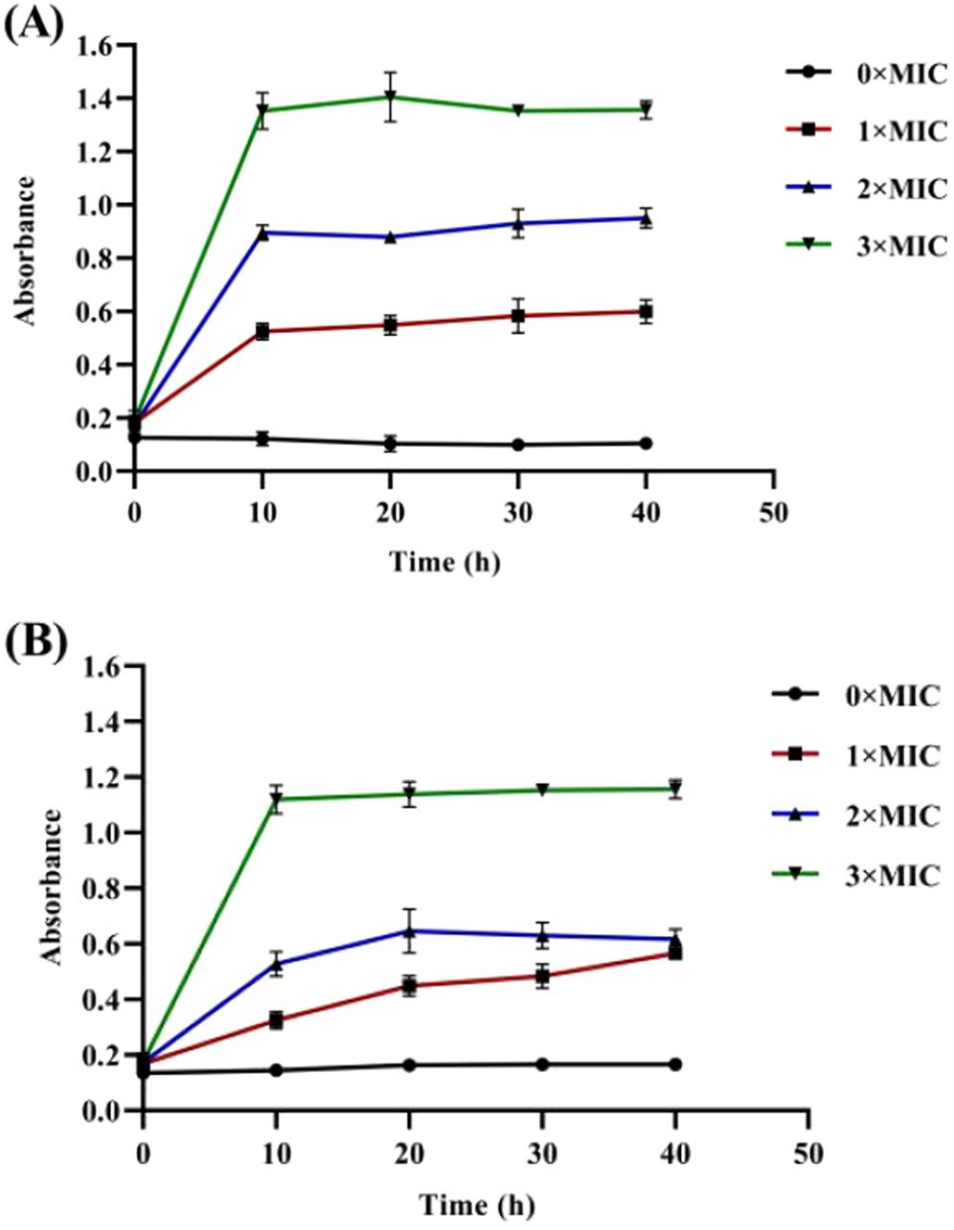
The effect of rhubarb ethanol extract at 1MIC, 2MIC, and 3MIC on the cell membrane permeability of *C. acnes* was examined analyzed at 260 nm. (A) The membrane permeability of strain 6919. (B) The membrane permeability of strain 11,827.

The cell membrane is an important part of the bacterial cell structure and serves an important barrier function. When bacteria are subjected to unfavorable growth conditions or antimicrobial agents, the cell membrane can be damaged, causing the leakage of large molecular substances (such as DNA, RNA, and so on) from inside the cells through the ruptured cell membrane (Nourbakhsh et al. 2022). Nucleic acids absorb light at 260 nm, and the extracellular leakage of nucleic acids leads to an increase in the absorbance of a bacterial suspension at 260 nm (Pimchan et al. 2023). Therefore, the integrity of the bacterial cell membrane can be assessed by measuring the absorbance of a bacterial suspension at 260 nm. As shown in Figure 2A,B, treatment with all three concentrations of the extract damaged the cell membrane of both strains, causing leakage of their contents into the culture medium. Moreover, higher extract concentrations caused more significant damage. The absorbance value at 260 nm of the blank control bacterial suspension did not significantly change during the 40 h incubation period. In contrast, the experimental groups presented a significant change in absorbance at 260 nm compared with that of the control group during the same incubation period. Initially, there was no obvious change in absorbance upon the addition of the extract; however, after 10 h of incubation, the absorbance of the suspension at 260 nm increased significantly and was positively correlated with the drug concentration. Thus, the rhubarb ethanol extract inhibited the growth of *C. acnes* by damaging the cell membrane, leading to the leakage of intracellular nucleic acids and other macromolecular substances. Compared with those in the control group, the bacterial suspensions in the experimental group presented a significant increase in absorbance. The increased absorbance at 260 nm suggests that the rhubarb ethanol extract induces the release of intracellular nucleic acids by compromising the bacterial cell membrane, which is likely a key mechanism by which the extract exerts its antibacterial effect. However, the possibility should be raised that the absorbance measured at 260 nm and fluorescent value also both reflect cell lysis and redundancy, therefore, this should also be considered as an explanation for the results (Molinaro et al. 2019).

### 5.2 Analysis of Bacterial DNA Damage

**Figure 3:**
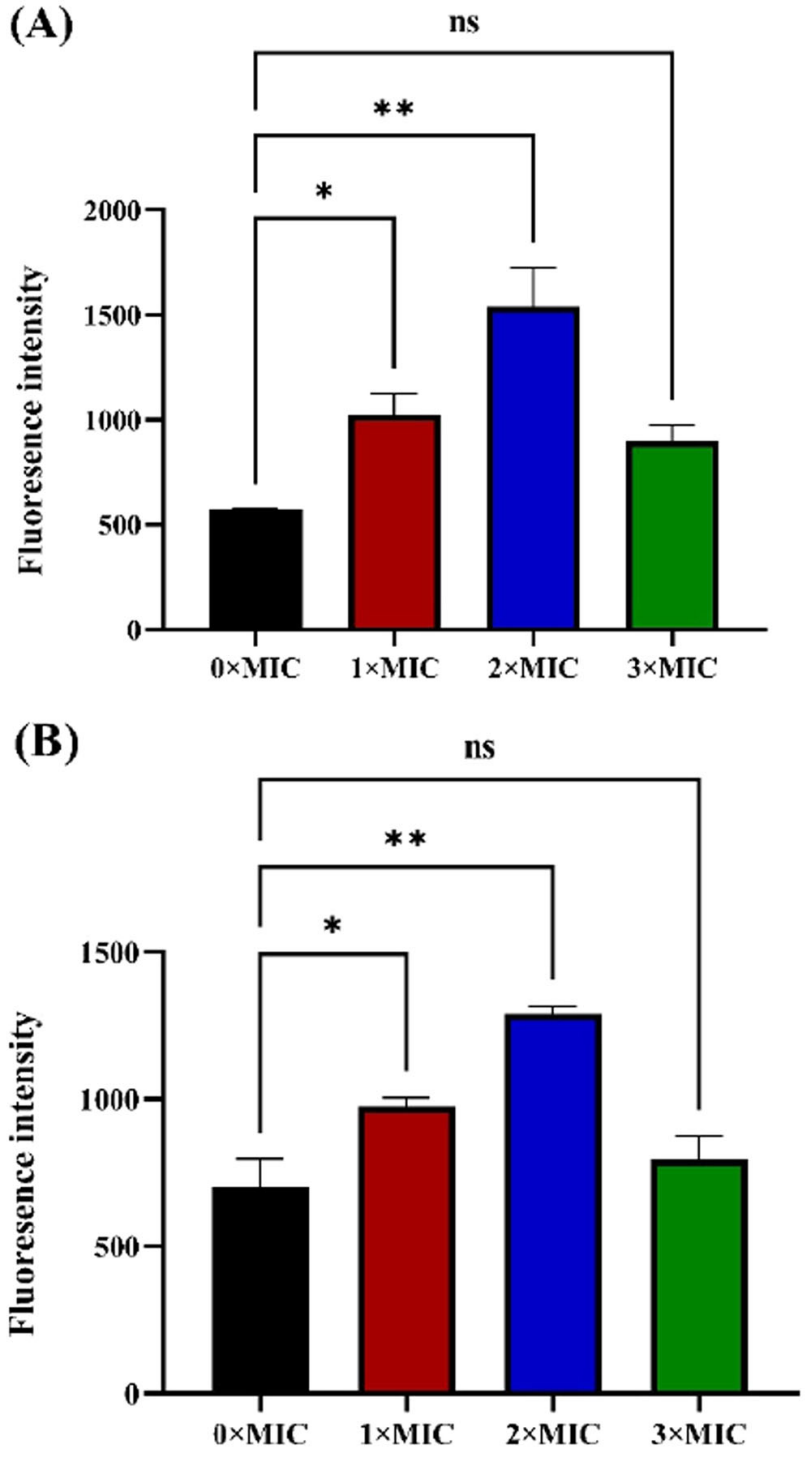
The analysis of DNA damage in *C. acnes* under rhubarb ethanol extract at 1MIC, 2MIC, and 3MIC concentrations was conducted. (A) DNA damage in strain 6919. (B) DNA damage in strain 11827. The excitation wavelength was 533 nm, and the emission wavelength was 615 nm. *Note:* Data are mean ± SD. Above each column, “*” indicate significant differences at *p* < 0.05, “**” indicate *p* < 0.01, “ns” indicate not significant.

The extent of damage caused by a drug can be inferred by staining bacterial nucleic acids with fluorescent dyes and measuring the fluorescence values using a fluorometer. PI is a nuclear dye that stains the DNA and RNA of dead cells, emitting red fluorescence. This dye stains the nucleic acids of dead bacteria only and not those of live bacteria, and the fluorescence intensity indicates whether a drug altered the permeability of the bacterial cell membrane, leading to apoptosis and nucleic acid leakage (Yan et al. 2023). As shown in Figure 3, the fluorescence intensity in the control group (0×MIC) was much lower than that in all the treated groups, indicating that normal DNA damage in bacteria occurred as expected. The fluorescence intensities in the experimental groups were notably greater than those in the control group, suggesting that the rhubarb ethanol extract had a significant antibacterial effect. Notably, for both strains 6919 and 11827, the fluorescence values at 2×MIC were the highest (2.57 and 1.84 times greater than those of the control), followed by those at 1×MIC (1.79 and 1.39 times greater than those of the control), which significantly exceeded those of all the other experimental groups (*p* < 0.05). However, although the fluorescence value for the 3×MIC group was still greater than that of the control group (1.57 and 1.14 times greater than that of the control), no significant difference was observed. This may be related to the lower bacterial density in the 3×MIC groups. According to the growth curve shown in Figure 1, the growth of both strains was noticeably weaker in the 3×MIC groups than in the other experimental groups, with a significant increase in the growth rate observed only after 30 h of culture. This led to a lower bacterial density, which may explain the lower fluorescence intensity in the 3× MIC group. The significant increases in fluorescence values in the treated groups compared with those in the control group highlight the extract's potent effect. The data revealed that both strains (6919 and 11827) presented peak fluorescence at 2×MIC, suggesting a threshold at which the antibacterial properties of the extract are maximized. The absence of significant differences at 3×MIC, despite its greater fluorescence intensity than that of the control, raises questions about potential bacterial adaptation which may need some time to adapt to the stimulation effect of the extract, or changes in density affecting the results (Purić et al. 2018). The growth curve analysis supports this hypothesis, indicating that the reduced growth in the 3×MIC group decreased the total nucleic acid concentration available for staining, thereby leading to misleading fluorescence intensity results. Further studies might explore the effects of extended exposure durations or different bacterial strains to enhance the understanding of the antibacterial mechanisms of the extract.

## 6 Antibiotic Resistance Reversal by the Rhubarb Ethanol Extract

### 6.1 Effects on Antibiotic Sensitivity After Subculture With the Rhubarb Ethanol Extract

#### 6.1.1 Effects on the MICs of Antibiotics After Passage With the Rhubarb Ethanol Extract

**Figure 4:**
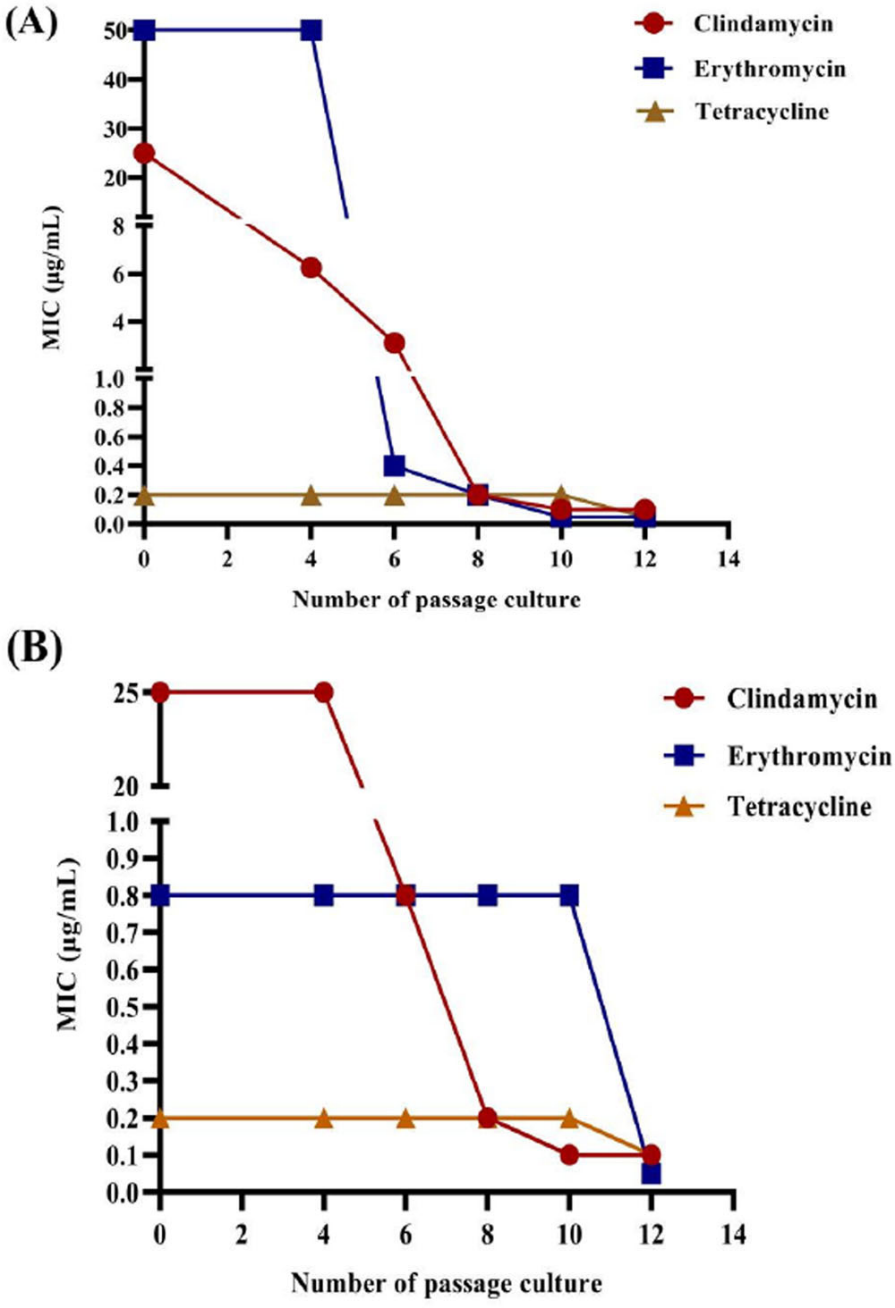
The effect on the MIC values of clindamycin, erythromycin, and tetracycline assessed after 0–12 passages of *C. acnes* cultured under the stress of ethanol extract. (A) Curve of 6919 and (B) Curve of 11827.

Rhubarb ethanol extract was added to the culture medium at a final concentration of 0.02 μg/mL, and passaging was performed every 48 h until the 12th generation. MICs of antibiotics used for the clinical treatment of acne (clindamycin, erythromycin, tetracycline hydrochloride, and tetracycline) were determined at generations 0, 4, 8, 10, and 12 to evaluate antibiotic sensitivity. As shown in Table 2 and Figure 4, the rhubarb ethanol extract significantly improved the sensitivity of *C. acnes* to clindamycin, erythromycin, and tetracycline but had no effect on tetracycline hydrochloride sensitivity. The MIC of clindamycin for ATCC11827 decreased from 25 to 0.1 μg/mL, resulting in a 250‐fold reduction in the MIC; that of erythromycin decreased from 0.8 to 0.05 μg/mL, that is, a 16‐fold reduction in the MIC; and that of tetracycline decreased from 0.2 to 0.1 μg/mL, that is, a twofold reduction in the MIC. For ATCC6919, the MIC of clindamycin decreased from 25 to 0.1 μg/mL, corresponding to a 250‐fold reduction in the MIC; that of erythromycin decreased from 50 to 0.05 μg/mL, corresponding to a 1000‐fold reduction; and that of tetracycline decreased from 0.2 to 0.05, corresponding to a fourfold reduction. Figure 4 shows the dynamic changes in the reduction in the MICs of the strains after passaging with clindamycin, erythromycin, or tetracycline.

**Table 2:** The MIC value of experiential antibiotics after subculture of 0–12 generations of strains (μg/mL).

Strain	Antibiotic	0	4	6	8	10	12
ACTT11827	Clindamycin	25.00	25.00	0.80	0.20	0.10	0.10
	Erythromycin	0.80	0.80	0.80	0.80	0.05	0.05
	Tetracycline	0.20	0.20	0.20	0.20	0.20	0.10
ACTT6919	Clindamycin	25.00	6.25	3.13	0.20	0.10	0.10
	Erythromycin	50.00	50.00	0.40	0.20	0.05	0.05
	Tetracycline	0.20	0.20	0.20	0.20	0.05	0.05

Thus, the above data indicated that there were no significant changes in antibiotic sensitivity from generations 0 to 4, but significant and noticeable changes occurred at generation 8, and much more significant effects were observed at generation 12. The impact of the extract on antibiotic sensitivity varied significantly among the different antibiotics. The greatest improvement was observed for erythromycin, which exhibited a 1000‐fold reduction in the MIC, followed by clindamycin, with a 250‐fold reduction. The reduction effect on tetracycline was much weaker, with a reduction of only twofold to fourfold, indicating a limited ability of the ethanol extract to reverse the sensitivity to antibiotics with lower initial MIC but a much more notable reversal effect for antibiotics with higher initial MIC.

#### 6.1.2 Effect on the MBCs of Antibiotics After Passage With the Rhubarb Ethanol Extract

Although the MICs of clindamycin and erythromycin greatly changed, the MBCs of these antibiotics did not significantly change during the experiment. However, interestingly, the MBC of tetracycline significantly changed. The MBCs of each generation of both strains in response to tetracycline are shown in Table 3. The MBCs of both strains decreased from 25 to 6.25 μg/mL when passaged with the rhubarb ethanol extract after the 8th generation, which also implies that the MBC was reduced fourfold. Therefore, compared with other antibiotics, the rhubarb extract improved the sensitivity of *C. acnes* to tetracycline to a greater extent, resulting in the strongest reversal potential in resistance.

**Table 3:** Effect on MBC values of *C. acnes* for tetracycline after passage (μg/mL).

MBC	0	2	4	6	8	10	12
11827	25	25	25	12.5	6.25	6.25	6.25
6919	25	25	25	25	6.25	6.25	6.25

Xin Ding et al. (Bao et al. 2020) reported that the administration of a polymer–antibiotic combination reversed the rifampicin resistance phenotype in *Acinetobacter baumannii*, resulting in a 2.5 × 10^5^‐fold reduction in the MIC and a 4096‐fold reduction in the MBC. This also enabled the repurposing of auranofin as an antibiotic against multidrug‐resistant (MDR) gram‐negative bacteria, with 512‐fold and 128‐fold reductions in the MIC and MBC, respectively. The results of this study revealed that the MIC was reduced by twofold to 1000‐fold, that is, a much weaker effect. This may be due to the use of different strains and drugs; however, both the approach in this study and the literature revealed that the experimental extract can reverse antibiotic resistance in MDR bacteria.

#### 6.1.3 Growth Curve Verification of the Reduction of MBC in *C. acnes*

**Figure 5:**
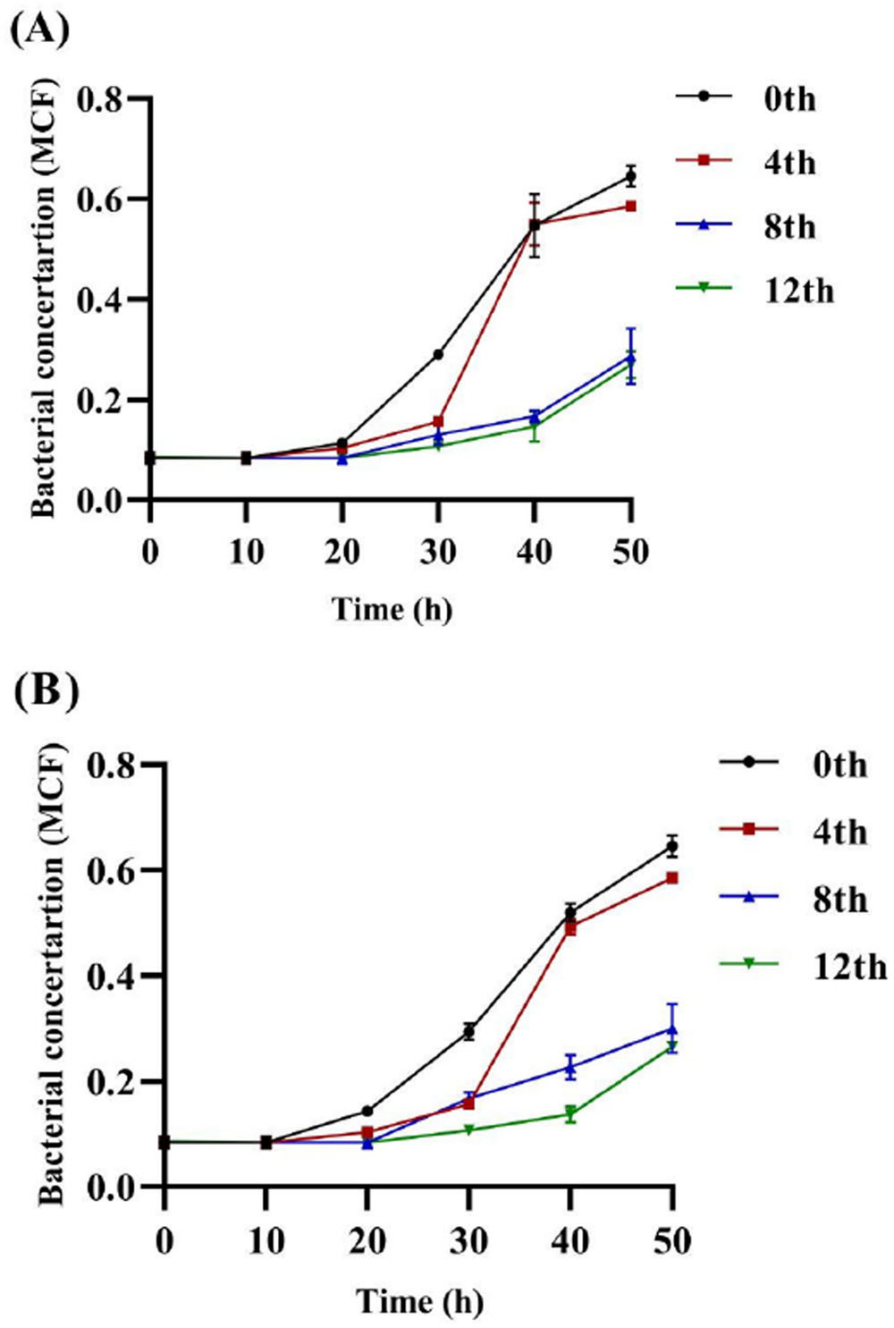
The dynamic growth curve of *C. acnes* in response to tetracycline at 0.5 MBC monitored over 0–12 passages with bacteria cultured under the stress of ethanol extract. (A) Curve of 6919, (B) Curve of 11827. *Note:* The bacterial concentration of MCF is McFarland Standard, which was measured by a McFarland concentration meter, where 1 MCF unit is equivalent to 3 × 10^8^ CFU/mL.

Tetracycline was chosen to verify the significant reversal of MBC in *C. acnes*. A concentration of 0.5×MBC was chosen for the verification study, and the growth curves of the strains were dynamically observed (Ding et al. 2020). As shown in Figure 5A,B, the growth curve of the 0th generation strain was significantly greater than that of the 4th, 8th, and 12th generations, and *C. acnes* growth decreased with increasing generations, with few significant differences between the 0th and 4th generations when the bacteria were cultivated after 50 h. However, a trend toward reduced growth was observed between the 4th and 0th generations. This finding implied that in the presence of constantly high concentrations of tetracycline, the bacteria presented increased sensitivity to the drug. Additionally, the growth of the 8th and 12th generations was highly significantly different from that of the 0th generation (*p* < 0.01). The 12th generation exhibited the slowest growth. These findings demonstrated that the rhubarb ethanol extract increased the antibiotic sensitivity of the experimental strains after cultivation under stress conditions. As the MBC is the minimum bactericidal concentration, when 0.5×MBC is used to provide stress to bacteria, it is clear from the later growth trends that the antibiotic sensitivity of the strain increases with continued passage, this is consistent with former results.

#### 6.1.4 Effects of Tetracycline on the ROS Levels in Subcultured Strains

**Figure 6:**
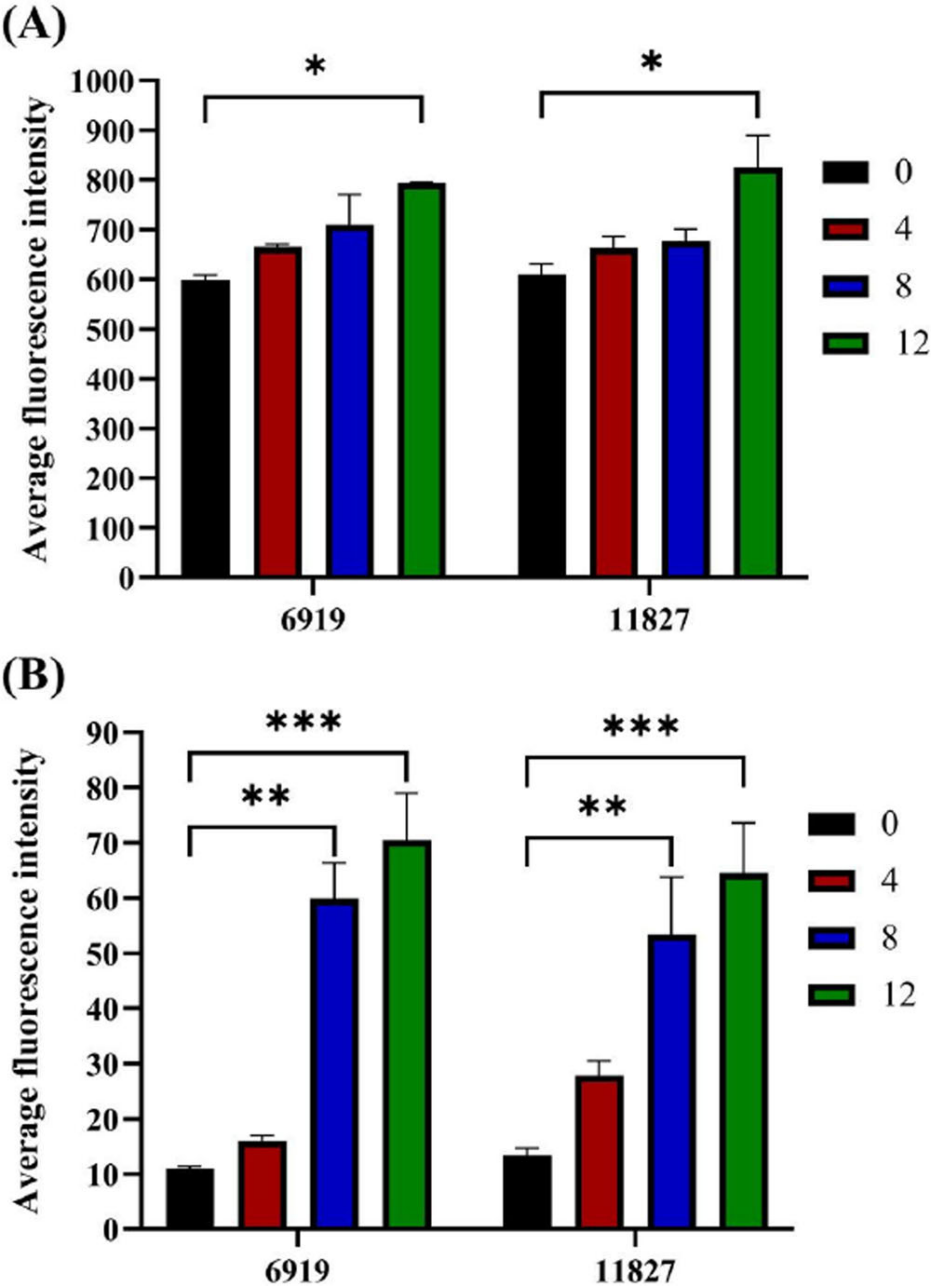
The ROS and DNA damage levels of subcultured strains (0–12 passages) affected by tetracycline were analyzed. (A) ROS level, (B) DNA damage level. *Note:* Data are mean ± SD. Above each column, “*” indicate significant differences at *p* < 0.05, “**” indicate *p* < 0.01, “***” indicate *p* < 0.001.

ROS are detected in bacteria primarily using the fluorescent probe DCFH‐DA, which can freely cross the cell membrane and enter bacterial cells. Once inside the cell, DCFH‐DA is hydrolyzed by intracellular esterases to form nonfluorescent DCFH. Since DCFH cannot cross the cell membrane, it remains inside the bacteria. In the presence of ROS, DCFH is oxidized to form DCF, which emits strong fluorescence. Thus, by measuring the fluorescence intensity of DCF, the level of ROS inside bacteria can be indirectly assessed. A relatively high ROS level is correlated with increased sensitivity of the bacterial strain to tetracycline. As shown in Figure 6A, the ROS level markedly increased in the 4th, 8th, and 12th generations of the *C. acnes* strains; the highest ROS levels were observed in the 12th generation and were significantly higher (*p* < 0.05) than the ROS levels in the 0th generation, indicating that the 12th‐generation strains are more sensitive to tetracycline. These findings also suggest that the rhubarb ethanol extract significantly enhanced the antibiotic sensitivity of *C. acnes*. Moreover, at the same concentration of tetracycline, the bactericidal effect of tetracycline significantly increased in the 12th‐generation strains. These findings suggest that prolonged exposure to the rhubarb ethanol extract progressively induces oxidative stress in *C. acnes*, which likely contributes to the increased sensitivity of these bacteria to tetracycline. The increased ROS production may be associated with the increased bactericidal effects of tetracycline, as evidenced by the significant improvement in antibiotic efficacy against the 12th‐generation strains.

#### 6.1.5 Effects of Tetracycline on DNA Damage in Subcultured Strains

PI is a nucleic acid dye that cannot penetrate the intact cell membrane, but in middle‐ and late‐stage apoptotic cells and dead cells, PI can penetrate the cell membrane and stain the nucleus red. The cells were fixed and stained with PI. Since PI specifically binds to cell DNA, the fluorescence intensity is well linearly correlated with the amount of PI binding (Supabowornsathit et al. 2022). The maximum absorption wavelength is 493 nm, and the maximum emission wavelength is 630 nm. In this study, the fluorescence value after PI staining was measured via Leica laser confocal microscopy, which can reflect the degree of drug damage to bacteria and the degree of cell apoptosis. As shown in Figure 6B, the fluorescence intensities of strains 6919 and 11827 of the 0th generation were lower than those of the subsequent generations, with the highest fluorescence intensities observed in the 12th‐generation strains. Compared with those of the 0th generation, no significant differences were observed in the fluorescence values of the 4th generation of both strains, whereas from the 8th generation onward, highly significant differences were noted (*p* < 0.01), with the 12th generation showing even more significant differences (*p* < 0.001). These findings provide evidence that the rhubarb ethanol extract significantly increased the sensitivity of *C. acnes* to tetracycline. These findings are consistent with earlier experimental results and further confirm that the rhubarb ethanol extract has the potential to reverse tetracycline resistance.

The above results indicate that the rhubarb ethanol extract significantly improved sensitivity to antibiotic in *C. acnes*. This effect was most pronounced for erythromycin and clindamycin, indicating that the extract may alter bacterial mechanisms related to antibiotic resistance, such as improving drug penetration or enhancing the efficacy of antibiotics through synergistic effects. The weaker effect on tetracycline suggests that the reversal mechanism may be more effective for antibiotics with a higher initial MIC value against the strain. Although there was no significant change in the MBC for clindamycin or erythromycin, a notable reduction in the MBC of tetracycline was observed, suggesting that the rhubarb ethanol extract enhanced the bactericidal effect of tetracycline. These findings suggest that the extract may alter the bacterial cell membrane or the intracellular environment, increasing bacterial susceptibility to antibiotics. This finding is consistent with the hypothesis that the extract can increase the bactericidal activity of tetracycline, particularly through oxidative stress. ROS production increased with each passage with rhubarb ethanol extract, with the highest levels observed in the 12th generation. The significant increase in ROS levels in later generations indicates that prolonged exposure to the rhubarb ethanol extract induces oxidative stress, which may contribute to the observed reversal of antibiotic resistance. These findings support the idea that oxidative stress plays a key role in sensitizing *C. acnes* to antibiotics, particularly tetracycline. The fluorescence levels revealed that the rhubarb ethanol extract caused significant damage to the cell membrane and increased nucleic acid leakage, especially in the 12th generation. The effects of the extract might be selective for certain antibiotics, possibly due to differences in mechanisms of action or resistance.

Therefore, the rhubarb ethanol extract effectively improved sensitivity to antibiotics in *C. acnes*, particularly for clindamycin, erythromycin, and tetracycline. The mechanism appears to involve the induction of oxidative stress, which enhances the bactericidal effects of these antibiotics. Future studies should explore the specific interactions between the rhubarb ethanol extract and various antibiotics to clarify why tetracycline hydrochloride resistance was unaffected. These findings suggest that future botanical drugs have excellent development potential for reversing antibiotic resistance.

#### 6.1.6 Chemical Composition of the Rhubarb Ethanol Extract

**Figure 7:**
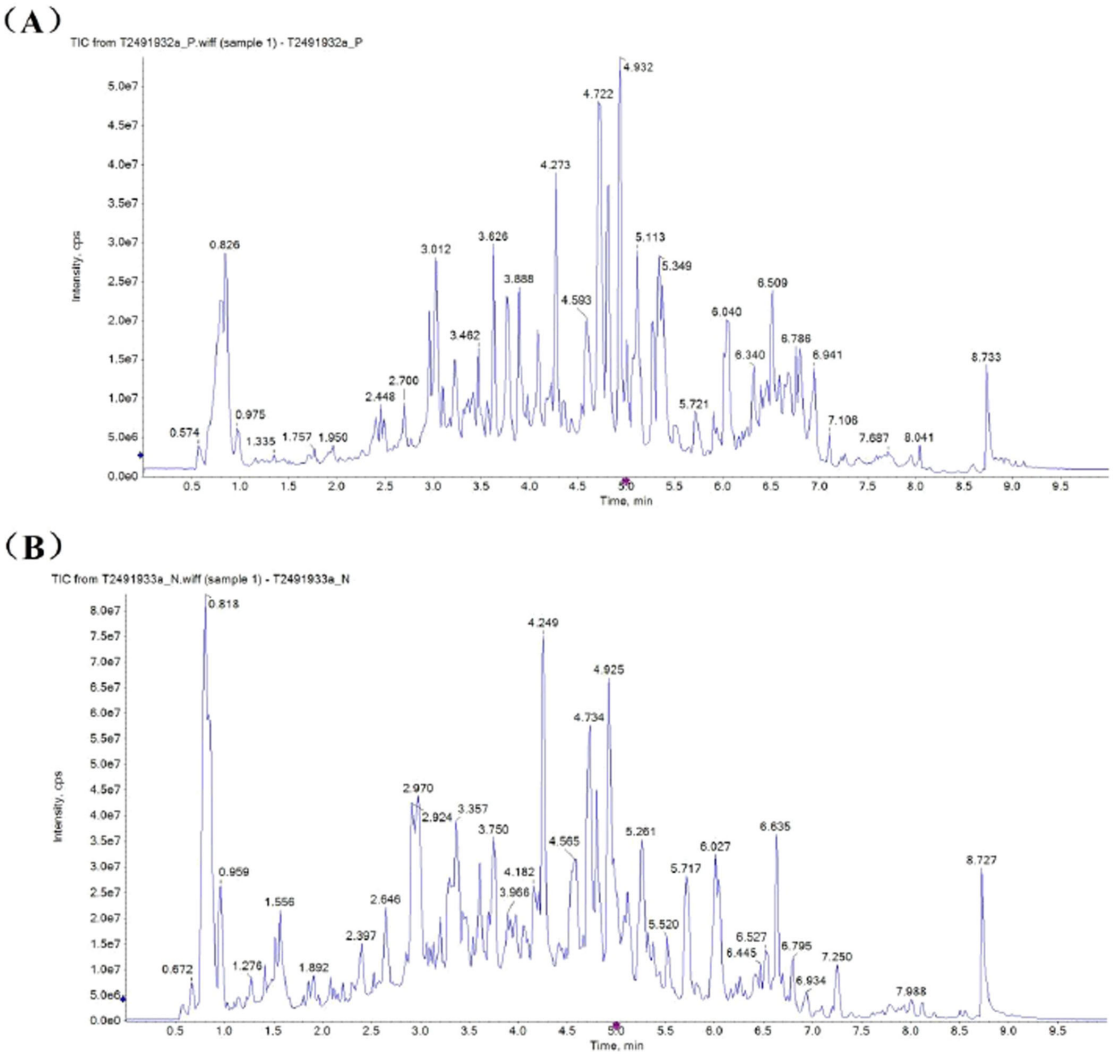
Graph of rhubarb ethanol extract of LC‐MS detection (A is for negative model, and B is for positive model).

To elucidate the main chemical components of the rhubarb ethanol extract, the extract was subjected to liquid chromatography‒mass spectrometry. A total of 50 compounds with high match scores and relatively high content ratios (over 0.1%) were identified in the samples, and the results are shown in Table 4 and Figure 7. The main components were flavonoids (10), heterocyclic compounds (8), benzene and its substituted derivatives (6), organic acids and their derivatives (5), aldehydes, ketones, esters (5), tannins (4) and others. Among them, the relative content of (‐)‐epicatechin was the highest, at 2.67%. In general, most of these compounds are catechins, anthraquinones, etc., which are the main active ingredients of rhubarb roots and exhibit good antibacterial and antioxidant activities. These compounds can reverse resistance, exert antioxidant effects and may cooperate to kill bacteria by blocking the efflux pumps of resistant bacteria at the same time (Boddu et al. 2024). Moreover, these components may be critical components that cause oxidative damage in bacteria, such as through ROS generation, during culture under stress conditions, affecting the activities of enzymes involved in drug resistance and reversing antibiotic resistance. As reported by Su, Ting et al. (E)‐anethole, anisyl acetone, anisyl alcohol and anisyl aldehyde, identified from extracts of *Illicium verum*, exhibit synergistic antibacterial activity against 67 clinical antibiotic‐resistant isolates (Su et al. 2020). These findings indicate that multidrug‐resistant bacteria may be less resistant to plant extracts than to antibiotics and may regulate bacterial enzyme activity and antibiotic metabolism through multiple chemical components, thereby exerting bacteriostatic effects and reversing antibiotic resistance (AlSheikh et al. 2020). Rhein methylate and emodin (0.59%) are both chemicals with antibacterial properties, they have been identified in the extract together, and their relative contents are approximately 2.18% (Jiang et al. 2022). These two compounds may act synergistically and likely contribute significantly to the observed antibacterial activity of the extract (Jiang et al. 2022). However, single antibiotic drugs do not have these advantages. Thus, research on botanical drugs has great future potential. The presence of naphtofluorescein and sodium dodecyl sulphate in the sample may be due to the utilization of pesticide and per‐treatment of plant growth in the field in agro‐industrial production (Zheltukhina et al.). Normal metabolites, like the detection of similar results of detection of naphtofluorescein in some plants (Jain et al. 2021, Zheltukhina et al. 2025).

**Table 4:** Chemical composition of rhubarb extract used in this study.

Compounds	Class I	Formula	Molecular weight (Da)	CAS	Relative ratios (%)
(‐)‐Epicatechin	Flavonoids	C_15_H_14_O_6_	290.08	490‐46‐0	2.67 ± 0.02
Naphthofluorescein	Heterocyclic compounds	C_28_H_16_O_5_	432.10	61419‐02‐1	2.17 ± 0.10
Rhein Methylate	Benzene and substituted derivatives	C_16_H_10_O_6_	298.05	69119‐31‐9	1.59 ± 0.05
Torachrysone 8‐O‐Glucoside	Aldehyde, Ketones, Esters	C_20_H_24_O_9_	408.14	64032‐49‐1	1.26 ± 0.08
Procyanidin B1	Tannins	C_30_H_26_O_12_	578.14	20315‐25‐7	1.19 ± 0.06
Trehalose	Carbohydrates and Its metabolites	C_12_H_22_O_11_	342.12	99‐20‐7	1.08 ± 0.13
Cosmosiin	Heterocyclic compounds	C_21_H_20_O_10_	432.11	578‐74‐5	0.92 ± 0.06
Cianidanol	Heterocyclic compounds	C_15_H_14_O_6_	290.08	154‐23‐4	0.91 ± 0.02
Procyanidin B3	Tannins	C_30_H_26_O_12_	578.14	23567‐23‐9	0.90 ± 0.02
Isomaltulose	Carbohydrates and Its metabolites	C_12_H_22_O_11_	342.12	58166‐27‐1 | 15132‐06‐6	0.83 ± 0.09
Nodakenetin	Heterocyclic compounds	C_14_H_14_O_4_	246.09	495‐32‐9	0.82 ± 0.13
Rubiadin	Benzene and substituted derivatives	C_15_H_10_O_4_	254.06	117‐02‐2	0.79 ± 0.03
Liquiritin	Flavonoids	C_21_H_22_O_9_	418.13	551‐15‐5	0.62 ± 0.00
Emodin	Benzene and substituted derivatives	C_15_H_10_O_5_	270.05	518‐82‐1	0.59 ± 0.03
7‐Hydroxyflavanol	Flavonoids	C_15_H_10_O_4_	254.06	492‐00‐2	0.53 ± 0.05
Arecatannin B1	Heterocyclic compounds	C_45_H_38_O_18_	866.21	—	0.53 ± 0.01
Acitretin	Organic acid and Its derivatives	C_21_H_26_O_3_	326.19	55079‐83‐9	0.48 ± 0.05
Melibiose	Carbohydrates and Its metabolites	C_12_H_22_O_11_	342.12	—	0.43 ± 0.03
Epicatechin‐(4beta‐> 8)‐epicatechin‐(4beta‐> 8)‐catechin	Flavonoids	C_45_H_38_O_18_	866.21	—	0.43 ± 0.03
Pterin	Heterocyclic compounds	C_6_H_5_N_5_O	163.05	2236‐60‐4	0.38 ± 0.01
Procyanidin B2	Tannins	C_30_H_26_O_12_	578.14	29106‐49‐8	0.37 ± 0.00
Kaempferol	Flavonoids	C_15_H_10_O_6_	286.05	520‐18‐3	0.36 ± 0.01
Procyanidin C1	Tannins	C_45_H_38_O_18_	866.21	37064‐30‐5	0.27 ± 0.01
Syringetin‐3‐o‐glucoside	Flavonoids	C_23_H_24_O_13_	508.12	40039‐49‐4	0.26 ± 0.001
Chrysophanol‐8‐O‐beta‐D‐(6’‐O‐malonyl)glucoside	Benzene and substituted derivatives	C_24_H_22_O_12_	502.11	205107‐13‐7	0.23 ± 0.07
1‐Hexadecanoyl‐2‐octadecanoyl‐sn‐glycero‐3‐phosphocholine	GP	C_42_H_84_NO_8_P	761.59	59403‐51‐9	0.22 ± 0.19
LPE(0:0/24:0)	GP	C_29_H_60_NO_7_P	565.41	—	0.22 ± 0.03
3,4‐Dimethoxycinnamic acid	Organic acid and Its derivatives	C_11_H_12_O_4_	208.07	14737‐89‐4	0.22 ± 0.00
Pyrazin‐2‐carboxylic acid	Organic acid and Its derivatives	C_5_H_4_N_2_O_2_	124.03	—	0.22 ± 0.02
Hispidol	Aldehyde, Ketones, Esters	C_15_H_10_O_4_	254.06	5786‐54‐9	0.21 ± 0.01
Ellagic acid	Benzene and substituted derivatives	C_14_H_6_O_8_	302.01	476‐66‐4	0.21 ± 0.01
Cynarine	Flavonoids	C_25_H_24_O_12_	516.13	30964‐13‐7	0.21 ± 0.02
3’‐Methoxydaidzein	Aldehyde, Ketones, Esters	C_16_H_12_O_5_	284.07	21913‐98‐4	0.19 ± 0.08
Baicalein	Aldehyde, Ketones, Esters	C_15_H_10_O_5_	270.05	491‐67‐8	0.19 ± 0.04
FAA(18:1)	FA	C_18_H_34_O_2_	282.26	506‐17‐2	0.18 ± 0.04
Marmesin	Heterocyclic compounds	C_14_H_14_O_4_	246.09	13849‐08‐6	0.18 ± 0.04
Ononin	Flavonoids	C_22_H_22_O_9_	430.13	486‐62‐4	0.18 ± 0.01
2‐(9h‐Carbazol‐9‐yl)benzoic acid	Organic acid and Its derivatives	C_19_H_13_NO_2_	287.09	6286‐88‐0	0.14 ± 0.01
2‐Benzylsuccinic acid	FA	C_11_H_12_O_4_	208.07	884‐33‐3 | 36092‐42‐9	0.14 ± 0.00
Moupinamide	Benzene and substituted derivatives	C_18_H_19_NO_4_	313.13	66648‐43‐9	0.13 ± 0.00
Z‐Ala‐asp‐OH	Amino acid and Its metabolites	C_15_H_18_N_2_O_7_	338.11	79458‐93‐8	0.13 ± 0.01
Sodium dodecyl sulfate	Others	C_12_H_25_NaO_4_S	288.14	151‐21‐3 | 12738‐53‐3 | 12765‐21‐8 | 1334‐67‐4	0.13 ± 0.01
Canrenone	Aldehyde, Ketones, Esters	C_22_H_28_O_3_	340.20	976‐71‐6	0.13 ± 0.03
Daidzein	Flavonoids	C_15_H_10_O_4_	254.06	486‐66‐8	0.12 ± 0.00
(‐)‐Epicatechin gallate	Heterocyclic compounds	C_22_H_18_O_10_	442.09	1257‐08‐5	0.11 ± 0.00
Icosa‐5,14‐dienoic acid	Organic acid and Its derivatives	C_20_H_36_O_2_	308.27	122055‐58‐7	0.10 ± 0.01
Lithocholic acid	Bile acids	C_24_H_40_O_3_	376.30	434‐13‐9	0.10 ± 0.00
14,15‐Leukotriene C4(ExC4)	GP	C_47_H_83_O_13_P	886.56	206059‐96‐3	0.10 ± 0.05
Homoplantaginin	Flavonoids	C_22_H_22_O_11_	462.12	17680‐84‐1	0.10 ± 0.01
Berberine	Alkaloids	C_20_H_18_NO^4+^	336.12	2086‐83‐1	0.10 ± 0.03

Therefore, in future research, it will be necessary to further examine how antibiotic metabolism and enzyme activity in bacteria are affected by plant extracts. Moreover, the specific active substances in the rhubarb ethanol extract still need further elucidation and verification. For example, active substances can be screened via surface plasmon resonance (SPR) screening on the basis of their binding to bacterial proteins. The conclusions obtained in this study provide an important basis for innovative acne treatment strategies.

## 7 Conclusion

Considering the urgent need for alternative therapies and previous clinical treatments involving the use of rhubarb for treating acne, the aim of this study was to investigate the inhibitory effects of the crude extract of rhubarb against *C. acnes*, with a particular focus on its potential to reverse antibiotic resistance. The results revealed that the screened rhubarb ethanol extract presented a significant antibacterial effect and notably enhanced the antibiotic sensitivity to *C. acnes* after serial culture with a low concentration of rhubarb ethanol extract. The rhubarb ethanol extract exerted potent antibacterial activity against *C. acnes* by disrupting cell membrane integrity and causing DNA damage in a clear dose‐dependent manner, which was also influenced by the induction of oxidative stress. In the experimental strains, the MIC of clindamycin decreased 250‐fold, that of erythromycin decreased 8‐ to 1000‐fold, and that of tetracycline decreased 2‐ to fourfold. The main components of the rhubarb ethanol extract with relatively high ratios were flavonoids, heterocyclic compounds, benzene and its substituted derivatives, and others. Among them, the relative content of (‐)‐epicatechin was the highest. But it should be noted that this study only focused on the MIC, MBC and related antioxidant indexes of the strains cultured under low‐dose drug stress and the tracking of the dynamic growth curve of the strains, although the initially observed MIC and MBC and the corresponding index surface experimental drugs may have the potential to reverse antibiotic resistance. However, further in‐depth mechanism and animal verification have not been carried out. Therefore, rhubarb ethanol extract may have potential for treating antibiotic‐resistant *C. acnes* infections if the findings of further in vivo research and clinical studies are in accordance with the results of this study. In all, this study offers new perspectives on the study of reversing resistance to antibiotics in *C. acnes* and provides a new reference for research on the potential of plant extracts in reversing antibiotic resistance.

## Author Contributions

**Doudou Yang:** design and experimental management, wrote the draft of the article and conducted key analysis. **Yu Cui:** experimental study, data collection and analysis, article revision. **Sen Zhu:** analysis of experimental data. **Ruoliang Wang:** data processing and analysis support. **Haijun Xu:** provided experimental materials. **Guanjie Zhao:** revision and improvement of the article. **Dandan Zhang:** revision and improvement of the article. **Yinku Liang:** design and experimental management, supervise the implementation of all experiments and fund support. All authors confirm that the content described in this article is true and accurate.

## Ethics Statement

The authors have nothing to report.

## Conflicts of Interest

The authors declare no conflicts of interest.

## Data Availability

The data that support the findings of this study are available from request of the corresponding author of Yinku Liang as listed in the manuscript.
